# Maternal High-Fat Diet Worsens Memory Deficits in the Triple-Transgenic (3xTgAD) Mouse Model of Alzheimer’s Disease

**DOI:** 10.1371/journal.pone.0099226

**Published:** 2014-06-11

**Authors:** Sarah A. L. Martin, Christine H. Jameson, Stuart M. Allan, Catherine B. Lawrence

**Affiliations:** Faculty of Life Sciences, University of Manchester, Manchester, United Kingdom; University of Cordoba, Spain

## Abstract

Alzheimer’s disease (AD) is not normally diagnosed until later in life, although evidence suggests that the disease starts at a much earlier age. Risk factors for AD, such as diabetes, hypertension and obesity, are known to have their affects during mid-life, though events very early in life, including maternal over-nutrition, can predispose offspring to develop these conditions. This study tested whether over-nutrition during pregnancy and lactation affected the development of AD in offspring, using a transgenic AD mouse model. Female triple-transgenic AD dam mice (3xTgAD) were exposed to a high-fat (60% energy from fat) or control diet during pregnancy and lactation. After weaning (at 3 weeks of age), female offspring were placed on a control diet and monitored up until 12 months of age during which time behavioural tests were performed. A transient increase in body weight was observed in 4-week-old offspring 3xTgAD mice from dams fed a high-fat diet. However, by 5 weeks of age the body weight of 3xTgAD mice from the maternal high-fat fed group was no different when compared to control-fed mice. A maternal high-fat diet led to a significant impairment in memory in 2- and 12-month-old 3xTgAD offspring mice when compared to offspring from control fed dams. These effects of a maternal high-fat diet on memory were accompanied by a significant increase (50%) in the number of tau positive neurones in the hippocampus. These data demonstrate that a high-fat diet during pregnancy and lactation increases memory impairments in female 3xTgAD mice and suggest that early life events during development might influence the onset and progression of AD later in life.

## Introduction

Alzheimer’s disease (AD) is a chronic progressive neurodegenerative disorder characterised by the accumulation of extracellular amyloid-beta (Aβ) plaques and neurofibrillary tangles composed of hyperphosphorylated tau [1]. AD patients present with complex cognitive impairment, with memory loss being one of the earliest clinical symptoms. Historically AD was thought to be a disease that developed in later life, but there is increasing evidence that the disease likely begins many years before clinical presentation. However, exactly when AD begins, and why some people get it and others do not, is not understood fully. Many environmental risk factors for AD (e.g. diet, hormones, metal exposure) are thought to have their major effects long before disease diagnosis [2], [3]. Obesity and consumption of a Western-style high-fat diet, especially in mid-life, are associated with an increased risk of dementia and AD in humans later in life [4]–[11]. The prevalence of AD is greater in countries where the intake of high-fat/high calorie diets is high, but lower in those that consume diets low in fat [12], [13]. Experimental animal studies also show that disease neuropathology (e.g. Aβ production) and/or behavioural deficits are enhanced in transgenic AD mice that are maintained on a high-fat diet [14]–[20].

As well as risk factors that occur in mid-life, evidence is now emerging to suggest that events as early as during fetal development may impact on the subsequent appearance of AD in late life [2], [3], [21]–[25]. The idea that the intra-uterine environment may influence the development of late-life chronic diseases was proposed over two decades ago. The Barker’s “fetal origins of adult disease hypothesis” states that environmental factors, especially nutrition, act in early life to program the risks for the onset of cardiovascular and metabolic disease in adult life [26], [27]. For example, high-fat diet-induced maternal obesity can impact on fetal growth, and subsequently increase the risk of offspring developing obesity in adulthood [28]–[30]. However, it has yet to be shown directly whether a change in environment, *in-utero* and early in development, can alter the onset and/or disease severity in transgenic AD models, although short-term exercise during pregnancy can decrease pathology in offspring of an AD mouse model [31].

Recent data from epidemiological studies show that maternal obesity is associated with an increased risk of childhood behavioural and cognitive disturbances [32], [33]. Furthermore, animals that are exposed to maternal obesity and high-fat diets during early development also exhibit altered behaviour including increased anxiety/stress, changes in locomotion, aggression, altered reward based behaviours and impaired cognitive function [34]–[42]. Thus, as obesity is a risk factor for AD and excess calories in adulthood worsen AD in animal models, it is possible that over-nutrition during pregnancy and lactation may affect the development of AD in offspring. In the present study we therefore tested the hypothesis that maternal over-nutrition due to high-fat consumption during pregnancy and lactation in an animal model of AD worsens and/or accelerates AD disease pathology and behaviour in offspring.

## Methods

### Ethics Statement

All experimental procedures using animals were conducted in accordance with the United Kingdom Animals (Scientific Procedures) Act, 1986 and approved by the Home Office and the local Animal Ethical Review Group, University of Manchester.

### Animals and Diets

Triple-transgenic AD (3xTgAD) mice (human APP_Swe_, PS1_M146_ and tau_P301L_) on a C57BL6/129sv background were supplied originally by Frank LaFerla and Salvadore Oddo (University of California-Irvine, CA, USA) and in-house colonies were established [43]. Mice were housed in standard housing conditions (temperature 20±2°C, humidity 55±5%, 12-hour light/dark cycle with lights on at 07.00am), and given *ad-libitum* access to standard rodent chow and water unless stated otherwise.

3xTgAD (8-week-old) females were mated with 3xTgAD males and once copulation was confirmed (by presence of vaginal plug), females were housed individually and randomly allocated to either a high-fat (60% fat by energy, 58G9, Test Diets, supplied by IPS Product Supplies Ltd, UK; *n* = 4) or a control diet (12% fat by energy; 58G7, Test Diets, supplied by IPS Product Supplies Ltd; *n* = 4), which were protein and micronutrient matched. All pregnant female mice (dams) were maintained on their respective diets throughout gestation and lactation and their body weight recorded. Once born, all offspring remained with their mothers during lactation until weaning at 21 days of age (3 weeks of age) when female mice were then placed on a standard chow diet (BK001, Special Diets Services, UK). There was no significant difference in litter size or ratio of male/female pups from control versus high-fat fed dams. Female offspring mice were maintained until 10 weeks (referred to as 2 months of age; control diet, *n* = 5; high-fat diet, *n* = 5) or 12 months of age (control diet, *n* = 5; high-fat diet, *n* = 5) when behavioural assessment was performed. Behavioural tests were also completed at 6 months of age in the mice maintained until 12 months of age. In all groups body weight and food intake were recorded weekly.

### Behavioural Tests

To evaluate memory and cognition, mice were subjected to the Y-maze spontaneous alternation test, and then either the novel object recognition or Morris water maze (MWM) test. To allow for habituation, home cages were placed in the testing room 30 min prior to testing. All behavioural observations were made between 10.00 am and 16.00 pm. The order of observation during this period was randomised across animals. No more than one behavioural test was completed during any single day. All equipment was cleaned with 70% ethanol between animals.

### Y-maze Spontaneous Alternation Test

Short-term working memory was assessed in the Y-maze spontaneous alternation test using a black opaque Perspex Y-maze with three arms (A, B and C), each containing a different visual cue. Each animal was placed in arm A of the Y-maze and allowed to explore for 8 min and arm entries made by each animal recorded. Arm entry was defined as having all 4 paws in the arm. Spontaneous alternation was defined as a successive entry into three different arms, on overlapping triplet sets in which three different arms are entered [44], [45]. The percentage of alternations was calculated as the number of alternations divided by the total arm entries minus two.

### Morris Water Maze

Spatial reference memory was assessed in the MWM using a 1.2 m diameter circular white opaque plastic tank containing water maintained at a temperature of 21–22°C and made opaque using water-soluble non-toxic white paint (Universe of Imagination, Geoffrey Inc., U.K.). Mice were initially given 2 d of visual platform training followed by 5 d of hidden platform training and a 1 d probe trial based on protocol by Vorhees and Williams [46]. Briefly, for the acquisition of the visual platform training, mice were placed into the maze without spatial cues, and allowed to locate a visual flagged platform. If the platform was not found within 2 min, the mouse was gently guided to it. Mice were given four trials each day for 2 d with a different start position and flagged platform location each trial. For the acquisition of the hidden platform task, four trials per day were conducted for 5 d. The sequence of start positions was different on each training day and visual spatial cues were located outside the tank. The latency to find the platform was recorded with a maximum of 2 min allowed. To test memory retention of the platform location, mice underwent a probe trial 24 h after the final hidden platform training trial. During the probe trial, the platform was removed and the mouse was placed in the pool and allowed to swim for 30 s. Time spent in each quadrant was recorded. Each trial was monitored and analysed using a CCTV tracking camera (Vista protos IV, UK) and 2020 PLUS tracking software (HVS Image, Buckingham, UK). The escape latency (sec) and swim speed (m/sec) during the hidden platform training, and the percentage time in the target quadrant and the average time spent in all other quadrants during the probe trial were calculated.

### Novel Object Recognition Test

Short-term non-associative memory based on the natural exploration of novelty in mice was assessed in the novel object recognition test. All mice were habituated to the circular arena for 5 min over 2 d. On the day of testing, mice were placed in the arena and allowed to explore two identical unfamiliar wooden objects (Universe of Imagination, Geoffrey, Inc., U.K.) for 10 min (phase 1). The objects were placed in the centre of the arena, 5 cm from the edge and 8 cm away from each other. Mice were then removed and one of the objects was replaced with a novel object that varied in shape and colour. The novel object was placed randomly in either the left or right position. After a delay of 1 h, mice were placed back into the arena and allowed to explore for a further 4 min (phase 2). All behaviour was recorded with a camera (Sanyo Xacti VPC-C4, SANYO Fisher, CA) and MP4 video-clips were converted to an AVI format using Pazera MP4 to AVI converter 1.3 (Pazera-Software, PL). The duration (sec) spent exploring the objects was then measured using Observer 5.0 software (Noldus, Wageningen, The Netherlands) with the investigator being blinded to the sex and treatment of the mice. Exploration was defined as the amount of time the animals spent with their nose pointing within 2 cm of the objects. The percentage time spent exploring the objects was calculated for phases 1 and 2.

### Immunohistochemistry

After all behavioural studies at approximately 2 months and 12 months of age, mice were weighed, anaesthetised using isoflurane (1.5–2.5% in O_2_) and intracardially-perfused with 0.9% saline. The liver, spleen and a gonadal fat pad were dissected and weights recorded. The brain was removed and immerse fixed overnight in 4% paraformaldehyde (Sigma-Aldrich, UK; in 0.1 M phosphate buffer (PB)), before cryoprotection in 30% sucrose (Fischer Scientific, UK) in 0.1 M PB at 4°C for 24 h. Coronal 30 µm brain sections were then cut on a freezing sliding microtome (from 0.38 to –3.88 mm relative to bregma according to the atlas of Paxinos and Franklin [47]. Immunohistochemistry for either Aβ or phosphorylated tau was then performed on free-floating sections. Briefly, endogenous peroxidase was removed before treatment in blocking solution (10% normal horse serum in PB/0.3% triton). Sections were then incubated at 4°C overnight with either a mouse monoclonal anti-human amyloid 6E10 (1∶3000, Covance-Signet Laboratories UK) for Aβ, or mouse monoclonal anti-human PHF-tau (AT8; 1∶1000, Autogen Bioclear, UK) for hyperphosphorylated tau. After washes in PB/0.3% triton, sections were treated for 2 h in a biotinylated horse anti-mouse IgG antibody (1∶500; Vector Laboratories Ltd., Peterborough, UK). Following washes (in 0.1 M PB), sections were immersed in avidin-biotin-peroxidase complex (ABC, Vector Laboratories Ltd) for 30 min, rinsed in 0.1 M PB and colour-developed using a 0.05% diaminobenzidine solution (in 0.01% H_2_O_2_). Sections were mounted onto gelatine-coated slides, dried, and coverslips were applied before viewing under a light microscope. The number of immunopositive cells in the hippocampus expressing tau was counted unilaterally, using a light microscope, and the average number of cells per section calculated. The Aβ plaque burden was determined throughout in the hippocampus. The plaque area was measured in each section and the plaque burden calculated as percentage plaque area (immunopositive area/total area used x 100). The investigator was blinded to genotype and treatment. As no Aβ plaques are present in the cortex or amygdala, and very few neurofibrillary tangles in the amygdala (with none in the cortex) in 12-month-old 3xTgAD mice, AD-like pathology was analysed in the hippocampus only.

### Statistical Analyses

Data are represented as mean ± standard error of the mean (SEM) and were analysed with SigmaStat. Body weight was assessed using a 2-way repeated measures ANOVA with Bonferroni’s post-hoc test. Evaluation of memory in the Y-maze spontaneous alternation test, novel object test, the probe test of the MWM, organ weights and pathology were examined using Student’s *t*-tests. MWM training was assessed between cohorts on individual training days and compared within cohorts between the first day of training and successive days of training to assess improvement over time via 2-way repeated measures ANOVA with Bonferroni’s post-hoc test. Statistical significance was taken at *P*<0.05.

## Results

### Effect of Maternal High-fat Diet on Bodyweight in 3xTgAD Dams and Pups

**Figure 1:**
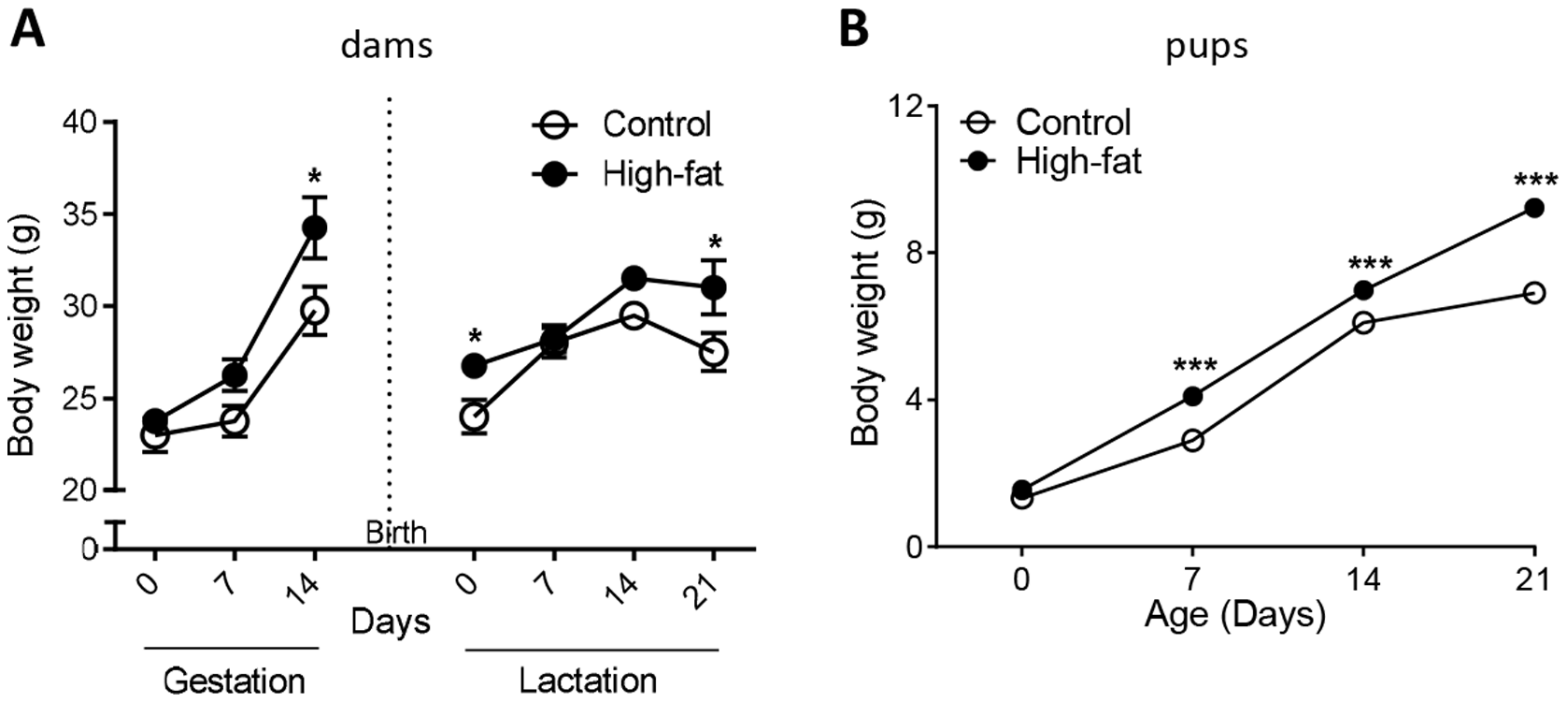
A high-fat diet during pregnancy and lactation increases bodyweight in 3xTgAD dams and offspring mice. Bodyweight (g) of female dams during pregnancy and lactation (A) and, offspring mice (of mixed sex) after birth and during lactation (B). Data are mean ± SEM for *n* = 4/group for dams and *n* = 23–30/group for pups. *P<0.05, ***P<0.001 versus control fed groups.

After confirmation of mating, 3xTgAD dams that were maintained on a high-fat diet weighed more than control fed mice at day 14 of gestation. Most dams gave birth at 18–19 days of gestation. After birth 3xTgAD dams fed a high-fat diet were significantly heavier than control fed dams at days 0 and 21 of lactation (Figure 1A).

At birth (day 0) and day 21 there was a significance increase in bodyweight of 3xTgAD offspring (unsexed) from dams fed a high-fat diet compared to control diet (Figure 1B).

### Effect of Maternal High-fat Diet on Bodyweight in 3xTgAD Offspring at 2 and 12 Months of Age

**Figure 2:**
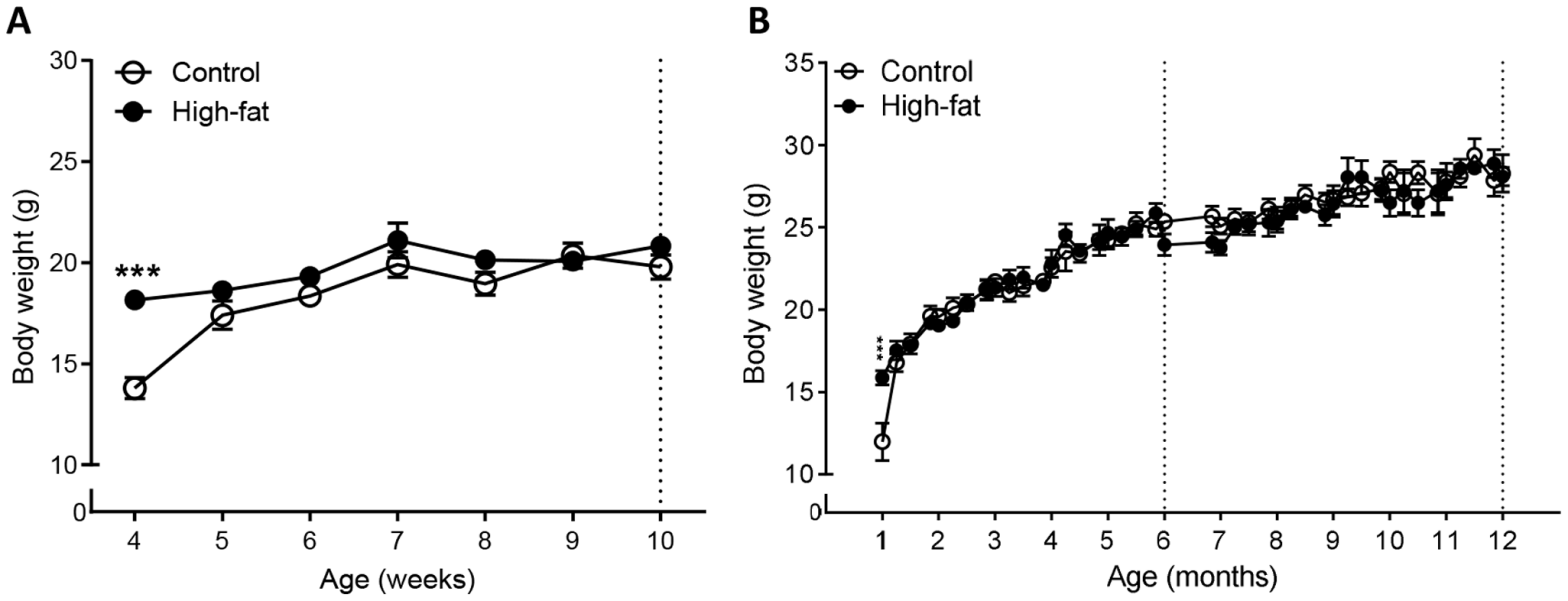
A high-fat diet during pregnancy and lactation transiently increases bodyweight in female 3xTgAD offspring. In separate groups of mice bodyweight was measured weekly after weaning between 4–10 weeks of age (approximately 2 months of age) (A) and 1–12 months (B) in female 3xTgAD offspring mice from either control or high-fat fed dams. Dashed line indicates the age at which behaviour was assessed (data are presented in Figures 3 and 4). Data are mean ± SEM for *n* = 5 per group. ***P<0.001 versus control fed mice.

In both cohorts of mice, at 4 weeks/1months of age, after weaning and sexing, female 3xTgAD offspring mice from high-fat fed dams weighed significantly more (31–33%) than control fed mice (Figures 2A and B). However after this time and up to 10 weeks of age (2 months, Figure 2A) and 12 months of age (Figure 2B) there was no difference in bodyweight between the two groups.

Between both 4–10 weeks and 1–12 months of age there was no significant difference in the average weekly food intake for female 3xTgAD offspring mice from high-fat compared to control fed dams (4–10 weeks: control, 20.7±0.9 g versus high-fat, 21.8±2.8 g, *p*>0.05; 1–12 months: control, 21.3±0.5 g versus high-fat, 21.2±0.4 g; *P*>0.05).

After the behavioural tests were performed in both cohorts (at approximately 2 or 12.5 months of age) there was a no significant difference in bodyweight and weight of the gonadal fat, liver and spleen between female 3xTgAD mice from dams fed a high-fat or control diet (Table 1).

**Table 1:** Body and organ weights of 3xTgAD offspring female mice from dams fed a control or high-fat diet.

Weight (g)	Control	High-fat
***2-month-old***		
**Body**	21.5±0.6	21.2±0.9
**Fat**	0.36±0.05	0.25±0.03
**Liver**	0.82±0.02	0.91±0.04
**Spleen**	0.12±0.01	0.11±0.01
***12-month-old***		
**Body**	28.3±0.4	27.6±0.7
**Fat**	0.45±0.02	0.40±0.04
**Liver**	0.96±0.03	1.09±0.08
**Spleen**	0.14±0.01	0.16±0.03

Body, fat (gonadal), liver and spleen weight were measured in separate groups of 2- or 12-month-old female 3xTgAD offspring mice from dams fed either a control or high-fat diet throughout gestation and lactation. Data are mean ± SEM, *n* = 5/group. Student’s *t* test.

### Effect of Maternal High-fat Diet on Memory in 3xTgAD Offspring at 2 Months of Age

**Figure 3:**
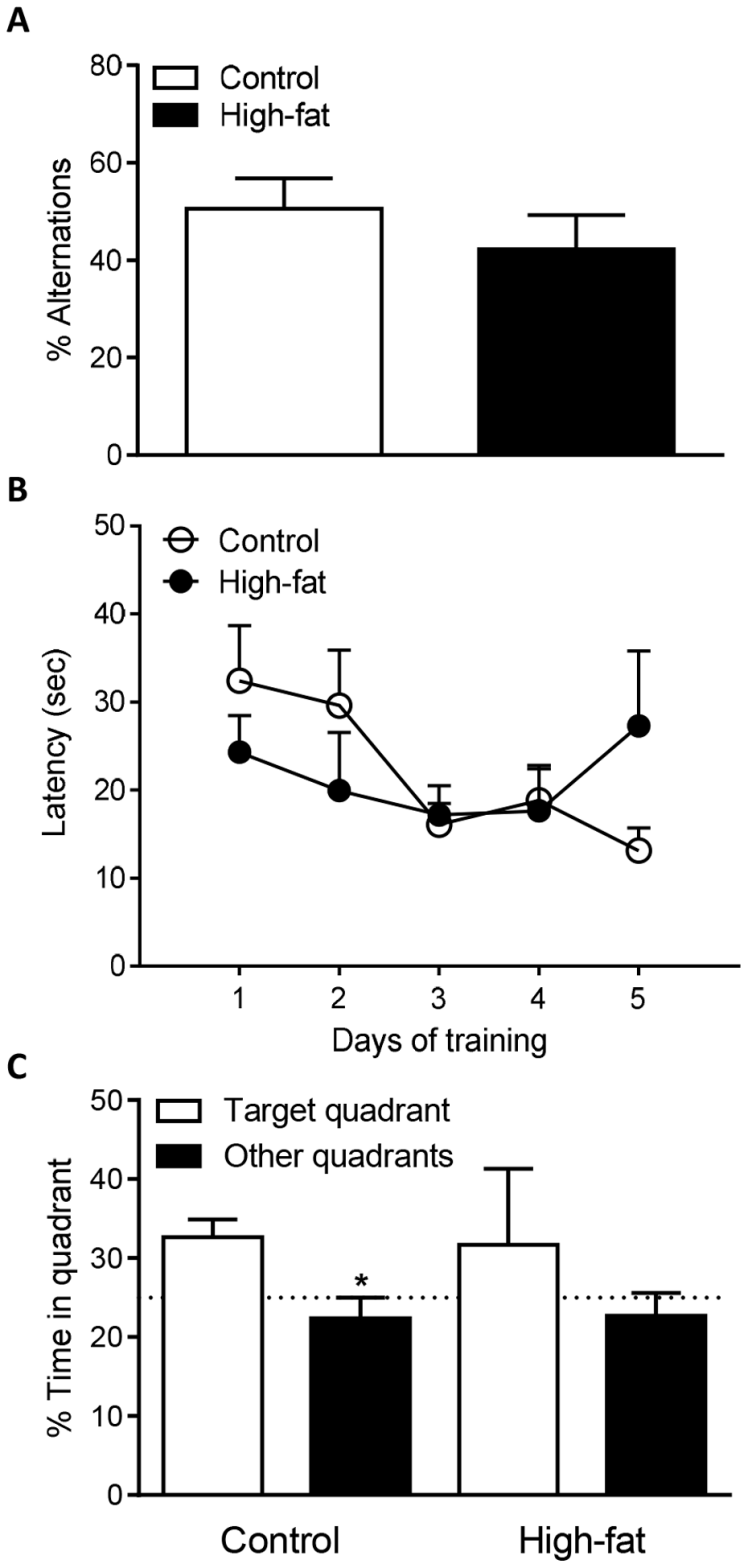
A high-fat diet during pregnancy and lactation impairs memory in female 3xTgAD offspring mice at 2 months of age. Memory was assessed in female 3xTgAD offspring mice using the Y-maze spontaneous alternation test (A) and the Morris water maze (MWM) test (B–C). For the MWM mice were given four trials a day for 5 d of submerged platform training (B). Twenty-four hours after the final trial mice were given a probe test with no platform (C). Data are mean ± SEM for *n* = 5 per group. *P<0.05 versus the target quadrant for control fed group.

At 2 months of age cognition was assessed in 3xTgAD female offspring mice from dams fed either a control or high-fat diet using the Y-maze and MWM tests. In the Y-maze, there was no significant difference in the % alternation between control and high-fat fed 3xTgAD offspring mice (Figure 3A). In the acquisition phase of the MWM, control- and high-fat fed groups of female 3xTgAD mice were unable to learn the location of the platform as no significant difference in latency was observed between days 1 and 5 of training (Figure 3B). No significant difference in swim speed was observed between control and high-fat fed 3xTgAD offspring mice (data not shown). In the probe test (of the MWM) female 3xTgAD mice from dams fed a control diet spent significantly more time in the target quadrant demonstrating memory formation, however a high-fat diet impaired memory as female 3xTgAD mice from high-fat fed dams spent the same amount of time in the target quadrant versus other quadrants (Figure 3C). Male 3xTgAD mice were also assessed but appeared to be cognitively less able than female mice. As early as 2 months of age all male mice had memory deficits in the acquisition and probe phase of the MWM test regardless of maternal diet (data not shown). No conclusion could therefore be made about the effect of maternal high-fat diet in male mice, since the mice already showed significant cognitive impairment.

### Effect of Maternal High-fat Diet on Memory in 3xTgAD Offspring at 6 and 12 Months of Age

**Figure 4:**
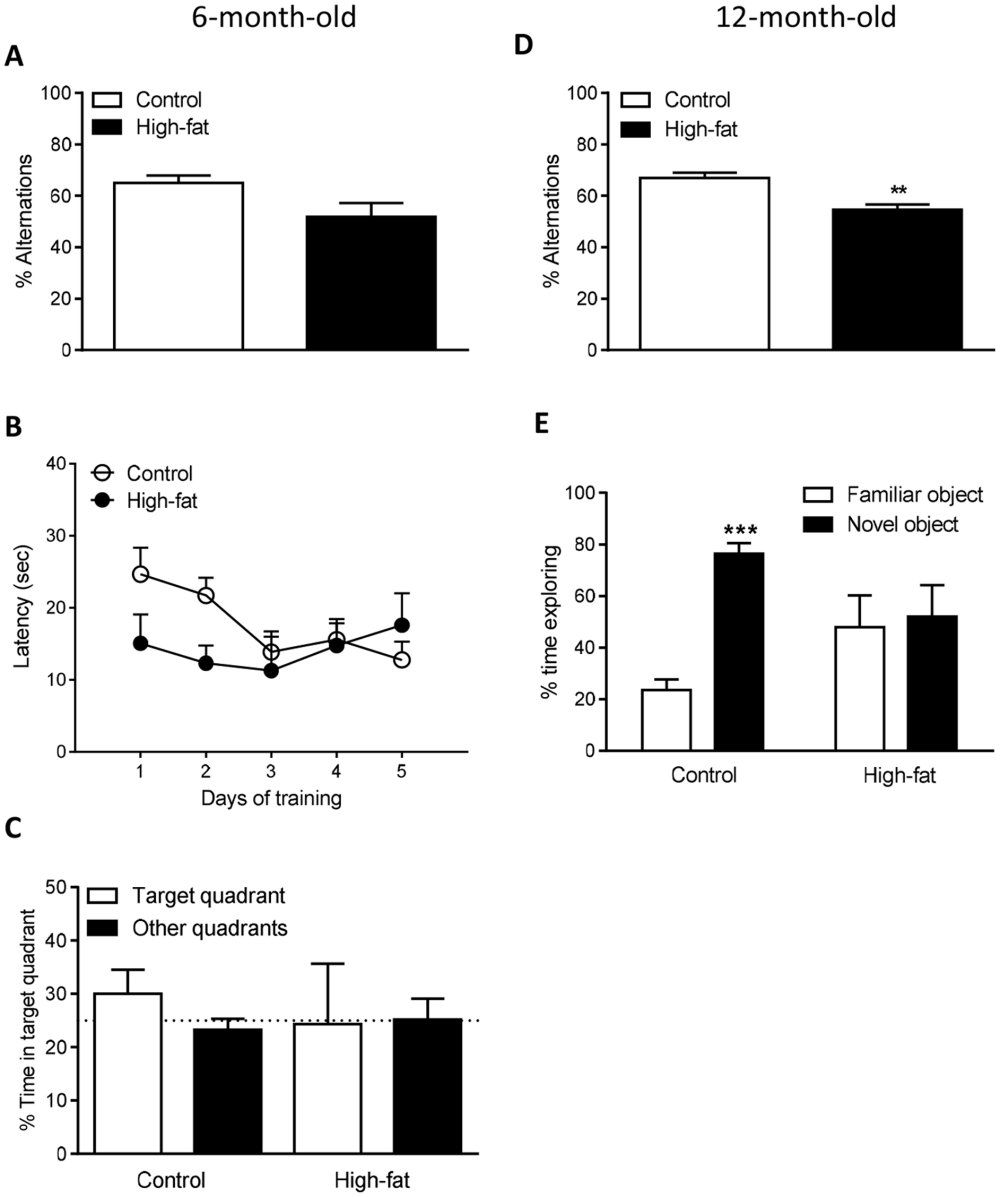
A high-fat diet during pregnancy and lactation impairs memory in female 3xTgAD offspring mice at 12 months of age. Memory was assessed in female 3xTgAD offspring mice using the Y-maze spontaneous alternation test (A) and the Morris water maze (MWM) test (B–C) at 6 months of age, and the Y-maze spontaneous alternation test (D) and novel object recognition test (E) at 12 months of age. For the MWM mice were given four trials a day for 5 d of submerged platform training (B). Twenty-four after the final trial mice were given a probe test with no platform (C). Data are mean ± SEM for *n* = 5 per group. **p<0.01 versus control fed group; ***P<0.001 versus familiar object in control fed group.

Cognition was assessed at both 6 and 12 months of age in the same groups of female 3xTgAD mice from dams fed either a control or high-fat diet. In the Y-maze at 6 months of age there was no difference in the % alternations between 3xTgAD mice from the control or high-fat fed groups (Figure 4A). In the MWM, female 3xTgAD mice from dams fed a control or high-fat diet did not learn as there was no significant difference in latency in the acquisition phase test between days 1–5 (Figure 4B) or the % time spent in the target quadrant versus other quadrants in the probe task (Figure 4C). There was also no significant difference in swim speed in the acquisition phase of the MWM between control and high-fat 3xTgAD female offspring mice (data not shown).

At 12 months of age the female 3xTgAD offspring from dams fed a high-fat diet were cognitively less able than control mice as significantly fewer % alternations were performed in the Y-maze in the female high-fat group compared to controls (*P*<0.01; Figure 4D). As all mice were not able to learn in the MWM test at 6 months of age (Figures 4B and C) this assessment was not repeated in 12-month-old mice but the novel object recognition test was performed. For 12-month-old female 3xTgAD mice from dams fed either a control or high-fat diet, there was no difference in exploration of identical objects during phase 1 of the novel object recognition test (data not shown). In phase 2 of the novel object recognition test, 12-month-old 3xTgAD female mice from control-fed dams exhibited intact memory as more time was spent exploring the novel versus the familiar object (*P*<0.001), whereas a maternal high-fat diet impaired memory as 3xTgAD mice from dams fed a high-fat diet spent a similar amount of time exploring both objects (Figure 4E).

### Effect of Maternal High-fat Diet on AD Pathology in 3xTgAD Offspring at 12 Months of Age

**Figure 5:**
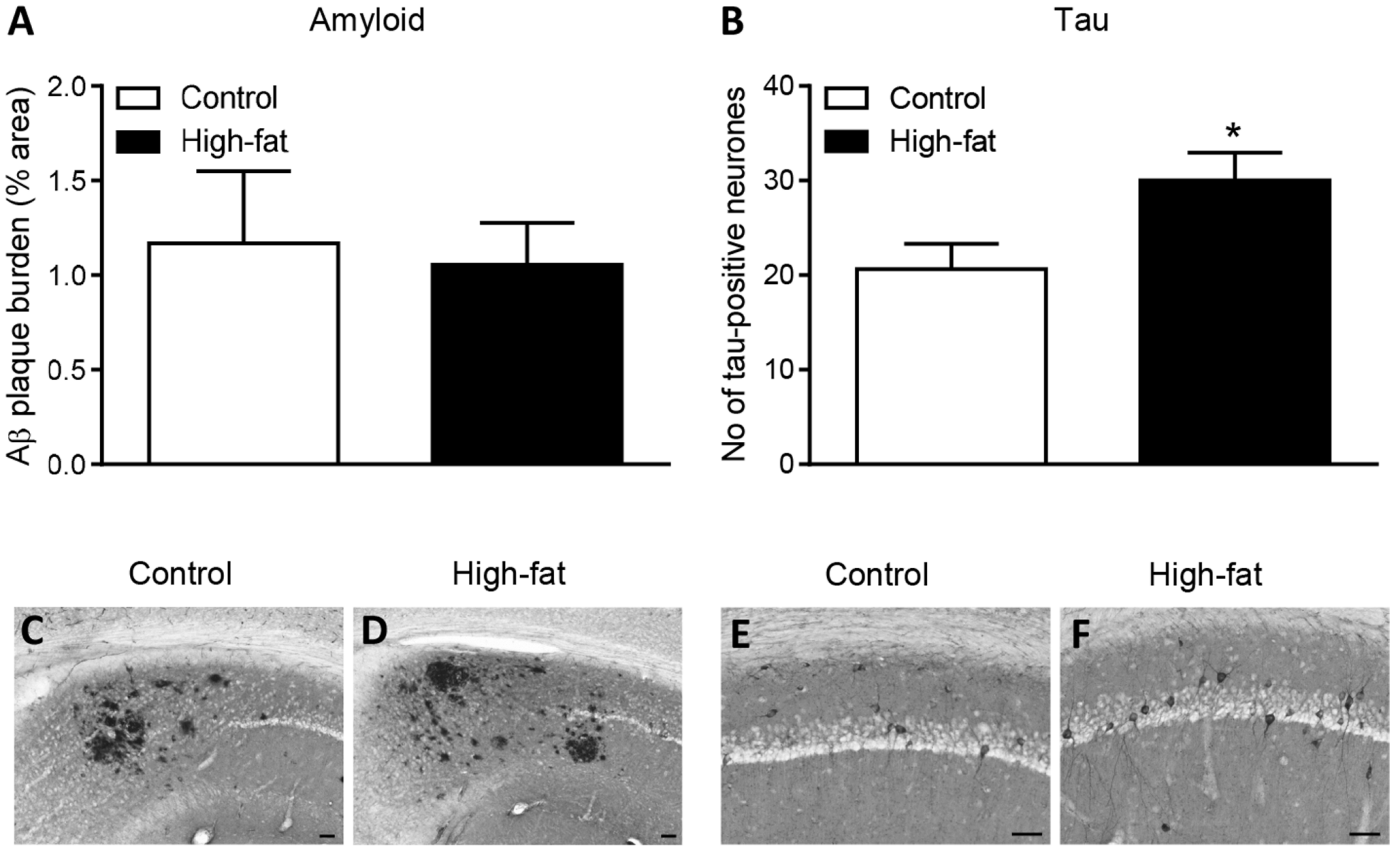
A high-fat diet during pregnancy and lactation increases tau pathology in the hippocampus of female 3xTgAD offspring mice at 12 months of age. The Aβ extracelluar plaque burden (A) and the number of hyperphosphorylated tau-positive neurones/section (B) were assessed in the hippocampus of female offspring mice from dams fed a control or high-fat diet during pregnancy and lactation. Data are mean ± SEM for *n* = 5 per group. *P<0.05. C-F are representative photomicrographs in the hippocampus of Aβ plaques (C–D) and hyperphosphorylated tau (E–F) detected by immunohistochemistry using 6E10 and AT8 antibodies, respectively. Scale bars: 100 µm.

At 12-months of age there was a significant increase (∼50%) in the number of phosphorylated tau-positive neurones in the hippocampus of 3xTgAD female mice from dams fed a high-fat diet compared to control diet (Figures 5B and E–F). There was no effect of a maternal high-fat diet on the Aβ plaque burden in the hippocampus of female 3xTgAD mice (Figures 5A and C–D). At 2 months of age no Aβ plaques or phosphorylated tau-positive neurones were detected in the hippocampus of 3xTgAD female offspring mice from dams fed a control or high-fat diet (data not shown).

## Discussion

The present study demonstrates that a high-fat diet during gestation and lactation affects memory and increases tau pathology in female offspring 3xTgAD mice. This study is therefore the first to demonstrate that a maternal high-fat diet can affect disease severity in an AD mouse model, although we previously reported that a post-natal high-fat diet can affect memory in male 3xTgAD mice [20]. The negative effect of a maternal high-fat diet on memory in female 3xTgAD offspring was detected as early as 2 months of age and was still present in 12-month-old mice, demonstrating long-lasting changes in memory.

Although the data in the present study show that a high-fat diet during intrauterine and early postnatal development can affect cognition in offspring from 3xTgAD mice it is not known whether the maternal diet is mediating its effects during pregnancy or lactation or both. However, it is likely that they are equally important as both are critical stages of development. As the dams had higher bodyweight during gestation and lactation it also remains to be determined if the effects of maternal diet on memory are due to maternal obesity or the high-fat diet *per se*.

In humans, maternal obesity and high calorie diets during pregnancy increase risk of developing obesity in offspring [28]–[30] and, obesity at mid-life is associated with an increased risk of dementia and AD later in life [4]–[6]. In the present study a maternal high-fat diet increased bodyweight in female 3xTgAD offspring. However, these effects were transient and there was no significant difference in bodyweight or adipose tissue mass between control and high-fat fed female 3xTgAD offspring at the time of behavioural testing. Thus, the detrimental effects of maternal high-fat diet on memory in female 3xTgAD offspring in the current study are likely to be independent of bodyweight/adiposity. Alternatively, it is possible that the transient increase in body weight early in life has long lasting effects that worsen AD progression in later life. Sustained increases in bodyweight have been observed in non-transgenic offspring rodents from high-fat fed dams, although data are not consistent in all studies [48], [49]. It is possible that 3xTgAD mice respond differently to a maternal high-fat diet as the metabolism, appetite and bodyweight of these mice is altered when compared to non-transgenic controls [50], [51]. In addition to obesity, perinatal over-nutrition can also increase the risk of developing insulin resistance/diabetes and hypertension in offspring [52], [53], all of which are risk factors for AD [54]. However, as these parameters were not assessed in the present study their role in the detrimental effect of maternal obesity on cognitive function in AD offspring remains to be determined.

While this study is the first to report an effect of maternal diet on memory in an AD mouse model, changes in cognitive function have been observed in non-transgenic offspring mice from dams fed a high-fat diet, although findings are inconsistent. A maternal high-fat diet has been shown to decrease memory formation in both male [38] and female [41] mice. This negative effect on cognition in male mice is transient as a deficit in memory performance is observed at 3–4 but not 10–11 weeks of age in offspring from dams fed a 32% high-fat diet [38]. The detrimental effect of maternal high-fat diet in female offspring mice on memory was observed at 8 weeks of age, but in this study mice were maintained on the high-fat diet after birth until behavioural analysis [41] and therefore the post-natal impact of a high-fat diet cannot be separated from effects during pregnancy and lactation. Studies in rats have shown that there is no effect of a high-fat diet (60%) during pregnancy and lactation on memory in male offspring at 20 weeks of age [42]. However, a maternal high-fat diet sensitised offspring to high-fat feeding in adulthood, as a reduction in memory was observed in 20-week-old male rats that were born from high-fat fed dams and subsequently maintained on a post-natal high-fat diet [42]. In contrast to the detrimental effects of maternal obesity on cognition, an increase in memory formation has been observed in 3-month-old male and female offspring rats from dams fed a 60% high-fat diet during pregnancy and lactation [55]. Overall, therefore, the effects of a maternal high-fat diet in non-transgenic offspring rodents appear complex and are likely to depend on several factors including, the exact composition of the diet, duration and timing of exposure to diet, the type of behavioural test employed, and when the behavioural analysis is performed.

The effect of a maternal high-fat diet on memory in female 3xTgAD mice observed in the current study could be due to alterations in epigenetic regulation of neural pathways and molecular mechanisms involved in learning and memory in the hippocampus since maternal obesity and a high-fat diet during development can alter the structure and functional plasticity of the hippocampus in non-transgenic offspring mice. Fewer neurones and abnormal dendritic differentiation of new neurones are detected in the hippocampus of non-transgenic offspring mice that were exposed to a high-fat diet during gestation and lactation, effects that are possibly due to a reduction in brain-derived neurotrophic factor (BDNF) expression [38], [56]. Whether these changes in the hippocampus are also observed in female 3xTgAD offspring mice from dams fed a high-fat diet remain to be established.

3xTgAD mice present with both Aβ plaques and neurofibrillary tangles composed of hyperphosphorylated tau [43]. The effect of maternal obesity on memory in female 3xTgAD offspring mice was not associated with a change Aβ plaque pathology but with an increase in the number of tau-positive neurones. It is possible therefore that an increase in tau phosphorylation may play a role in the memory impairment observed in female high-fat fed 3xTgAD offspring. In support, high-fat diets during adulthood increase tau pathology in 3xTgAD mice [16] and also in other AD and tau-expressing mouse models [57]–[59]. These effects of a high-fat diet on tau might be due to a change in free fatty acids (FFA) profile, as saturated FFA, which are associated with obesity and high-fat diets, can increase tau phosphorylation in cortical neurones *in vitro* [60]. However, no effect of a high-fat diet is observed on Aβ and tau pathology in 3xTgAD male mice when the diet was given after the weaning period (from 8 weeks of age) [20].

In summary, these data demonstrate for the first time that maternal obesity in an experimental mouse model of AD can impair cognitive function in female offspring and imply that AD pathogenesis might be influenced during development. The idea that changes in maternal metabolism during a critical period of development (gestation and lactation) can impact on AD is supported by the demonstration that short-term exercise during pregnancy decreases AD pathology in offspring mice [31]. Thus, understanding the mechanisms involved in the detrimental effect of maternal obesity on AD progression will be of future importance given the prevalence of obesity in pregnant woman is increasing.
